# Combining immunotherapy with radiotherapy for glioblastoma: recent advances and challenges

**DOI:** 10.3389/fonc.2025.1523675

**Published:** 2025-05-22

**Authors:** Yuanyang Sun, Yun Wang, Shukun Mu, Xiaofeng Wu, Suchun Yu, Zhongming Wang

**Affiliations:** ^1^ School of Health Science and Engineering, University of Shanghai for Science and Technology, Shanghai, China; ^2^ Department of Radiation Oncology, Shidong Hospital, Yangpu District, Shidong Hospital Affiliated to University of Shanghai for Science and Technology, Shanghai, China; ^3^ Department of Pharmacy, Shidong Hospital, Yangpu District, Shidong Hospital Affiliated to University of Shanghai for Science and Technology, Shanghai, China

**Keywords:** glioblastoma, immunotherapy, radiotherapy, tumor microenvironment, clinical trial

## Abstract

Glioblastoma (GBM) is an extremely aggressive brain tumor. Its standard treatment currently involves surgery followed by radiotherapy and temozolomide. However, recurrence is frequently unavoidable, and the effect of various treatments is not ideal due to numerous inherent obstacles. Immunotherapy has demonstrated promising prospects in the management of various cancers. Despite several preclinical studies have shown that immunotherapy may improve the survival in GBM mouse model, the results from completed clinical trials reveal that it brings only limited benefit for GBM patients to date. Interestingly, several studies have demonstrated that radiotherapy not only eliminates tumor cells by inducing DNA damages but also improves the effect of immunotherapy by modulating immune response. Combining immunotherapy with radiotherapy for GBM has been evaluated extensively. Herein, we present the immunotherapy applied in GBM and highlight the importance of tumor microenvironment in immunotherapy for GBM. Moreover, we review the preclinical and clinical data for applying immunotherapy combined with radiotherapy for GBM. Finally, we also discuss the challenges facing combined treatment of immunotherapy and radiotherapy for GBM and further research aspects in the discussion section.

## 1 Introduction

Glioblastoma (GBM) generally has a poor prognosis with a median overall survival (mOS) of under 2 years and a 5-year survival rate of only 10% (1). The standard treatment for GBM includes surgery followed by concurrent radiotherapy combined with chemotherapy (2). However, the effect of this treatment is not satisfactory. Currently, immunotherapy has been investigated extensively in many malignant cancers (3–5). Immunotherapy has revolutionized the tumor treatment and brought new hope for the management of GBM. However, the BBB and immunosuppressive tumor microenvironment limit significantly the effect of immunotherapy for GBM (6). Radiotherapy is a common approach to treating various tumors. There is substantial evidence indicating that radiotherapy can exert systemic antitumor effect by enhancing the immune activation in many tumors (7–9). Several preclinical studies also reported that immunotherapy can exert radiosensitizing effect by normalizing tumor blood vascular system and alleviating hypoxia (10). A meta-analysis compared the effect and safety of radiochemotherapy alongside with immunotherapy against radiochemotherapy alone in newly diagnosed GBM (11). Nine clinical trials were included in this analysis. The results showed that immunotherapy was safely combined with radiochemotherapy. Nonetheless, combined treatment does not improve the survival greatly. Even so, combining immunotherapy with radiotherapy is still being actively researched in GBM (12, 13). Herein, we introduce the current situation of immunotherapy in GBM and biological rationale of combining immunotherapy with radiotherapy. Moreover, we summarize the recent progress of combined treatment of immunotherapy and radiotherapy for GBM and discuss certain current challenges that need to be addressed.

## 2 Immunotherapy

Immunotherapy for tumor treatment refers to utilizing immune system to identify and kill tumor cells. Currently, available immunotherapies for GBM treatment mainly consist of immune checkpoint inhibitors (ICIs), adoptive cell therapy (ACT), vaccine, oncolytic virus (Ov), and cytokine therapy (14, 15).

### 2.1 ICIs

Tumor cells generally employ immune checkpoints to evade the destructive impact of immune system (16). ICIs can inhibit this immunosuppressive effect and exert antitumor effect by blocking these immunosuppressive immune checkpoints. Pembrolizumab is a common ICI (anti-PD-1) for GBM. The outcomes of a clinical study revealed that giving neoadjuvant pembrolizumab, along with continued adjuvant treatment post-surgery, improved the mOS and mPFS in recurrent GBM, compared to those who only undergone adjuvant pembrolizumab (17). The mOS for neoadjuvant group and adjuvant group were 13.7 and 7.5 months, respectively. And the mPFS for neoadjuvant group and adjuvant group were 3.3 and 2.4 months, respectively. The neoadjuvant pembrolizumab enhanced the antitumor immune response both locally and systematically. Another phase II trial evaluated the effect of combining pembrolizumab with bevacizumab in contrast to administering pembrolizumab monotherapy in recurrent GBM (rGBM) (18). However, the outcomes showed that pembrolizumab, whether administered alone or alongside bevacizumab, had good tolerance but brought only modest benefits. Nivolumab is another anti-PD-1 antibody for treating GBM. A exploratory phase I trial assessed the tolerance of nivolumab, either alone or combined with ipilimumab, in rGBM (19). The outcomes revealed that the tolerance of nivolumab monotherapy was superior to that of dual therapy. The subsequent phase III trial compared nivolumab to bevacizumab in rGBM (20). The mOS for nivolumab group (9.8 months) was similar to that of bevacizumab group (10.0 months). The PFS and ORR for bevacizumab group were superior to those for nivolumab group. Another phase II trial evaluated the effect of neoadjuvant nivolumab in resectable GBM (21). The outcomes showed that no obvious clinical benefit was observed following surgery. Moreover, other immune checkpoints, such as CTLA-4 (22), CD47 (23), CD73 (24), and TIGIT (25), were also investigated in several preclinical studies or clinical trials.

### 2.2 ACT

ACT focuses on infusing immune cells to treat tumors, including genetically engineered T cells and tumor-infiltrating lymphocytes (TILs) (26). CAR T-cell therapy, which is a genetic engineering technique, adjusts T cells of patients to produce CARs aimed at tumor-associated proteins. There are preclinical evidence indicated that the CAR T-cells targeting IL-13Rα2 (27), EphA2 (28), HER2 (29), EGFR (30), and EGFRvIII (31) improved the survival in mouse GBM model. The results of clinical trials utilizing CAR T-cells for GBM also are promising. A phase I trial investigated the effect of the CAR- T cells targeting HER2 (VSTs) for progressive GBM (32). The outcomes showed that VSTs were well tolerated and brought clinical benefit for these patients. Among 16 patients, 1 patient exhibited a PR, 7 patients maintained SD and 8 patients progressed following VSTs infusion. Another trial evaluated the effect of CAR- T cells targeting IL13Ra2 for GBM (33). Reported results revealed that noticeable reduction of tumor lesions was observed, accompanied by increased cytokines and immune cells. Moreover, the CAR T-cells targeting EGFRvIII have also shown clinical benefit for GBM patients (34). Adoptive transfer of TILs is another common ACT, it has been demonstrated to lead to lasting regression in many tumors. A trial evaluated the safety of infusing autologous TILs and recombinant interleukin-2 locally for GBM. Six GBM patients received the infusion of TILs and recombinant interleukin-2 along with chemotherapy following surgery (35). The infusion of autologous TILs and recombinant interleukin-2 showed good therapeutic effect in treating GBM with acceptable toxicity. One patient showed a CR, two patients showed a PR, and three patients passed away due to progressive disease. No notable complications were observed in all patients. The clinical trial (NCT00331526) explored the effect of lymphokine-activated killer (LAK) cells for GBM (36). Thirty-three patients received adjuvant intralesional LAK cells therapy. LAK cells were well tolerated. The 1-year survival rate was 75%, with a mOS of 20.5 months. These outcomes demonstrated the potential of TILs transfer for GBM.

### 2.3 Vaccine

Several vaccines have been used to treat various tumor, with peptide and dendritic cell (DC) vaccines primarily used for treating GBM (37, 38). EGFRvIII, a deletion mutation of EGFR, can promote tumor development and enhance therapy resistance (39). A clinical study of rindopepimut (vaccine targeting EGFRvIII) plus bevacizumab for EGFRvIII-expressing rGBM indicated that rindopepimut plus bevacizumab brought potential PFS benefit (40). The 6-month PFS for rindopepimut cohort and control cohort were 28% and 16%, respectively. Another clinical study evaluated the effect of rindopepimut plus temozolomide in EGFRvIII-positive GBM (41). However, rindopepimut plus temozolomide did not improve the survival in contrast to temozolomide monotherapy. The mOS for rindopepimut plus temozolomide group and temozolomide group were 20.0months and 20.1months, respectively.

DC vaccine is another common vaccine in treating GBM. When activated by DCs, immune cells traverse BBB and reach tumor site to exert their antitumor functions (42). Moreover, DCs can promote the conversion of immunologically cold tumor into immunologically hot tumor (43). A research compared the OS for patients with nGBM and rGBM who underwent DCVax-L plus SOC vs external control patients who underwent SOC (44). Among 331 patients, 232 received DCVax-L, while 99 were given placebo. After experiencing recurrence, 64 patients from the placebo cohort switched to undergo DCVax-L. DCVax-L plus SOC improved the survival of GBM patients in contrast to external control patients. The mOS for 64 rGBM patients who underwent DCVax-L and control patients were 13.2 months and 7.8 months, respectively. The mOS for 232 nGBM patients who underwent DCVax-L and control patients were 19.3 months and 16.5 months, respectively.

### 2.4 OV therapy

OVs are genetically modified viruses that have a low level of pathogenicity and enhance the antitumor effect while sparing normal cells (45). The purposes for genetic modification of Ovs include (1): deleting virulence genes to enhance the safety (2), improving targeting to tumor cells (3), improving the effects against tumors. OVs can self-replicate inside tumor cells, directly destroying tumor cells. They also can activate immune responses by producing damage-related molecular patterns (DAMPs) and TAAs (46). Moreover, they can specifically suppress glioma stem cells (GSCs) that are crucial in promoting therapy resistance and tumor blood vessels formation (47). A clinical study of PVSRIPO (a nonpathogenic polio-rhinovirus chimera) for rGBM revealed that the 1- and 2- years survival rates of patients receiving PVSRIPO were superior to that of historical controls (48). Another clinical trial evaluated the effect of G47Δ (a oncolytic herpes simplex virus) for GBM following radiotherapy and temozolomide in Japan (49). The 1-year survival rate was 84.2%, with a mOS of 20.2 months. Due to the encouraging outcomes, G47Δ obtained temporary approval for GBM patients in Japan.

### 2.5 Cytokine therapy

Cytokines can improve immune response through adjusting the proliferation and differentiation immune cells (50). Several cytokines have been investigated as potential treatment to treat GBM, such as IFN-α, TGF-β, IL-2, IL-12, etc. A clinical trial investigated the efficacy of combining temozolomide with IFN-α compare to temozolomide monotherapy in nGBM (51). Temozolomide plus IFN-α greatly improved the mOS compared to temozolomide monotherapy. The mOS for patients receiving temozolomide in combination with IFN-α group was 26.7 months, significantly exceeding the standard group of 18.8 months. Another study investigated the effect of combining L19TNF (an antibody-conjugated cytokine) with CCNU (a chemotherapy drug) in mouse glioma model. L19TNF combined with CCNU exhibited strong anti-tumor effect. They discovered that L19TNF combined with CCNU led to tumor necrosis related to treatment, while also adjusting the immune microenvironment. Subsequently, they conducted a clinical trial for GBM patients who experienced first progression following chemotherapy and radiotherapy (52). The outcomes showed that L19TNF combined with CCNU was well tolerated. Common adverse events included symptomless rise in liver function text results and reduction in the counts of white blood cells and platelets. Among 6 patients, 1 showed a CR 9 months following treatment. And 1 showed a PR, with an 83% reduction in tumor lesions after 15 months. Other patients progressed ultimately at a distant site. The mPFS for all patients was 43.3 weeks, which is a significant improvement compared to the 4**–**12 weeks reported for CCNU alone. Increasing evidence demonstrated that cytokines may be a potential strategy for treating GBM.

## 3 Tumor microenvironment

**Figure 1:**
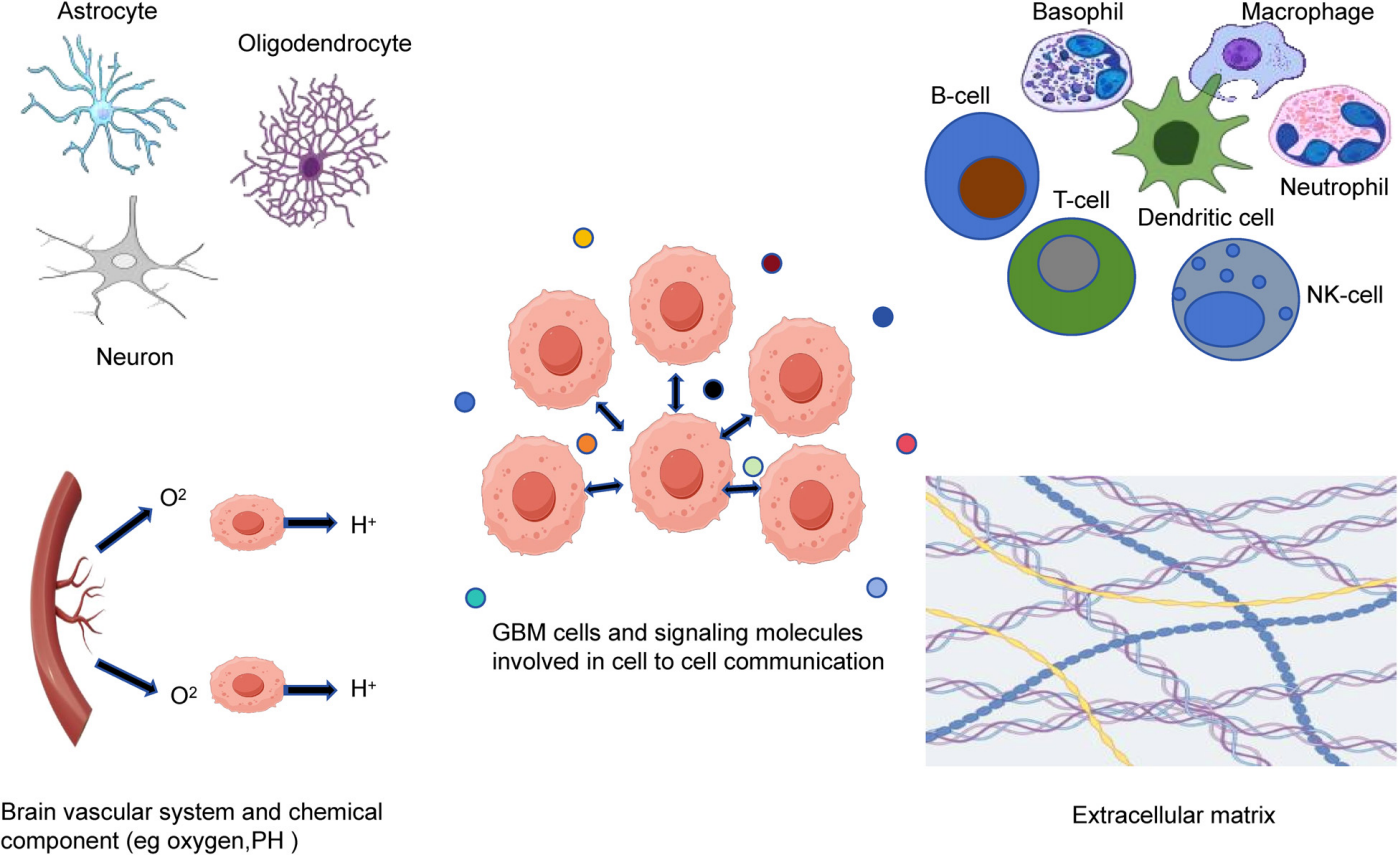
The tumor microenvironment components of GBM. Tumor microenvironment of GBM is a complex and heterogeneous system that consists of glioma and glioma stem cells, nervous system, immune cells, brain vascular system, extracellular matrix, signaling molecules and chemical components.

Tumor microenvironment of GBM is intricate and heterogeneous, consisting of various components (**Figure 1**). The main components of tumor microenvironment of GBM include the glioma and glioma stem cells, nervous system, immune cells, signaling molecules, extracellular matrix, perivascular niche, and several chemical components (53). GBM is marked by a strongly immunosuppressive microenvironment, resulting in therapy resistance and tumor recurrence. The immunosuppressive microenvironment is caused by various factors, including the increase in immunosuppressive cells and immunosuppressive cytokines, low tumor-infiltrating lymphocyte density and high expression of inhibitory immune checkpoint molecules (54). Tregs can modulate immune homeostasis by exerting immunosuppressive effect. Tregs can be attracted to tumor microenvironment through various cytokines, increasing their viability and expansion and then contributing the immunosuppression (55). Glioma-associated macrophages and microglia (GAMs) also is essential for the microenvironment of GBM, influencing the tumor growth, spread and recurrence. GAMs can be categorized into M1 phenotype and M2 phenotype. M2 phenotype contributes to the immunosuppressive microenvironment by producing IL-10 and TGF-β. It also facilitates GSCs proliferation. GSCs can enhance resistance by regulating cell metabolism in tumor microenvironment in addition to facilitating the tissue remodeling by reprogramming related cells (56).

The tumor microenvironment of GBM appears to induce radioresistance (57). Several evidence has demonstrated that GSCs can obstruct chemotherapy and radiotherapy through various mechanisms, thereby developing resistance (58). The percentage of glioblastoma cells expressing CD133, which is a marker for GSCs, rises following radiotherapy. CD133-positive GSCs can activate DNA damage checkpoint proteins when exposed to radiation. This allows them to repair DNA damage caused by radiotherapy more efficiently, consequently promoting their radioresistance (59). GSCs also can contribute to radioresistance by overexpressing the PCNA-associated factor (PAF). This factor can help control how accessible DNA translesion synthesis enzymes are to PCNA. Following GSCs are irradiated, PAF interacts with PCNA to produce TLS Pol η, which restores error-free DNA synthesis, subsequently promoting the proliferation of GSCs and enhances their radioresistance (60).

## 4 The immunostimulatory effects of radiotherapy

Besides providing local control, radiotherapy also can exert systemic effect on distant tumor lesions that have not been irradiated, which known as the abscopal effect (61). Numerous works have demonstrated that abscopal effect is closely related to immune system (62). Moreover, immunotherapy may boost abscopal effect induced radiotherapy (63). Radiotherapy can induce systemic immune responses, providing the rationale for combining radiotherapy with immunotherapy. Radiotherapy can exert powerful antitumor immune responses by influencing nearly every stage of tumor-immunity cycle (64). These effects mainly involve promoting the release and presentation of tumor antigens, enhancing the activation of immune cells, increasing TILs density, aiding T cells in recognizing tumor cells and augmenting antitumor effect. Moreover, radiotherapy can modify tumor microenvironment by generating certain cytokines and causing stromal, immunological, and vascular changes (65).

Radiation is the primary trigger for immunogenic cell death (ICD) that can promote the elimination of target cells by effector T cells and activation of antigen-presenting cells (APCs). The production of TAAs and DAMPs is a significant feature of ICD (66). DCs are the most effective APCs due to their unique dendritic morphology, which is essential in connecting innate and adaptive immunity. When DCs are exposed to radiation, it promotes significantly the production of chemokines CCL19 and CCL21. These chemokines subsequently attach to CCR7, facilitating DCs movement. The release of TAAs induced by radiotherapy promotes the activation of DCs and T cells. DAMPs mainly consist of calreticulin, high-mobility group protein box 1, and ATP (67). Radiotherapy can promote the translocation of calreticulin to the cell membrane and then enhance the phagocytosis of tumor cell by DCs (68). High-mobility group protein box 1 can activate DCs to trigger targeted T-cell responses (69). ATP can bind to P2X7 receptors on DCs, activating DCs and IFNγ-producing CD8-positive T cells (70).

Radiotherapy can cause the release of cytosolic DNA, which will be detected by STING triggering immune responses by producing IFN-I (71). Cytosolic DNA induces structural alterations in the cGAS enzyme, resulting in the production of cGAMP and then activating STING. Upon activation, STING dimers move from endoplasmic reticulum to Golgi apparatus and microsomes. STING activates the kinases TBK1 and IKK, subsequently phosphorylating IRF3 and IκB family and activating NFκB. The simultaneous activation of IRF3 and NFκB can induce IFN-I generation and inflammasome activation. IFN-I can regulate DC function, activate T cells, enhance NK cell cytotoxicity, and generate long-lived memory cells (72).

Radiotherapy can transform “cold” into “hot” tumor through various mechanisms. The release of TAAs caused by radiotherapy makes them more detectable by immune systems (73). DNA damages induced by radiation also can increase the tumor mutational load, favoring immune recognition and elimination (74). In general, tumor cells can avoid the clearance by immune system by reducing MHC-I expression. Radiotherapy-induced DNA damage can upregulate MHC-I expression and boost TAAs presentation by activating mTOR. This process ultimately makes tumor cells more vulnerable to attacks from the immune system (75). ICD induced by radiotherapy can overcome immune evasion by promoting the expression of CXCL10 and CXCL16. It also can enhance the tumor blood vessels extravasation, which results in an increased tumor-infiltrating lymphocyte density in tumor tissue. Radiotherapy also is essential in expanding TCR repertoire and increasing CD8/CD4 ratio, primarily by increasing the CD80 costimulatory signal (76). NK cells can identify and destroy tumor cells. Radiotherapy can boost NK cells activity through FAS and FAS ligand, as well as ADCC (77).

The low-dose radiation promotes tumor vascular system normalization and the transformation of M2-type macrophage into M1-type iNOS+ phenotype (78). The iNOS+ macrophages can trigger Th1 chemokines generation, attracting T cells to tumor tissues and then enhancing anti-tumor effects. Radiotherapy also can induce oxidative stress and DNA damages in surrounding tumor tissues that have not been directly irradiated, a phenomenon referred to radiation-induced bystander effect. This bystander effect is mediated by microparticles derived from irradiated tumor cells and exhibits a unique and broad spectrum of antitumor effects. Tumor cells ingesting microparticles are easily sequestered by activated TAMs, which makes tumor cells susceptible to destruction by immune cells.

## 5 Combining immunotherapy with radiotherapy for GBM

### 5.1 Preclinical data

The preclinical studies of combination treatment of immunotherapy and radiotherapy for GBM mainly focused on the ICIs combined with radiotherapy, and a small number of other immunotherapies in combined with radiotherapy. Herein, we review the preclinical studies of combining various immunotherapy agents with radiotherapy for GBM. Zeng et al. (79) investigated the effect of anti-PD-1 combined with stereotactic radiosurgery (SRS) in a mouse GBM model. Combining anti-PD-1 with SRS improved greatly the survival by enhancing the tumor infiltration of cytotoxic T cells and reducing Tregs, compared with either treatment alone. 4-1BB (CD137), an antibody against co-stimulatory molecules, can facilitate CD8+ T cell proliferation and pro-inflammatory cytokines production when it interacts with specific ligand (80). A study of combining 4-1BB (CD137) activation with anti-CTLA-4 and focal radiotherapy in mice intracranial GBM models revealed that this combination treatment led to an increased TILs density, improving significantly the survival of intracranial GBM model (81). TIM-3, a negative immunomodulator, is upregulated in GBM similar to PD-1 (82). Kim et al. (83) investigated the effect of anti-TIM-3 monotherapy or combined with anti-PD-1 and SRS in mice GBM models. The results revealed that triple therapy improved greatly the survival compared to anti-TIM-3 monotherapy. Moreover, the GBM mice that received the combination of anti-CD47 and radiotherapy or temozolomide also improved greatly the survival in contrast to those that undergone monotherapy (84). In addition to ICIs, combining peripheral vaccination and radiotherapy also has been demonstrated to improve antitumor effect in the management of GBM by promoting MHC-I expression and increasing T-cells infiltration (85).

### 5.2 Clinical data

Many clinical studies focused on combining immunotherapy with radiotherapy for GBM, especially involving ICIs (**Table 1**). The common immunotherapy agents used in combination treatment for GBM clinical trials mainly include durvalumab (anti-PD-L1), atezolizumab (anti-PD-L1), avelumab (anti-PD-L1), pembrolizumab (anti-PD-1), nivolumab (anti-PD-1), and ipilimumab (anti-CTLA-4). Herein, we review the completed clinical trials of combining immunotherapy with radiotherapy for GBM according to different immunotherapy agents.

**Table 1:** Clinical trials involving the combination of immunotherapy and radiotherapy for GBM.

Trial ID	Phase	Status	Conditions	Enrollment	Treatment approach
NCT00458601	II	Completed	nGBM	82	Rindopepimut (CDX-110) + Radiotherapy + Temozolomide
NCT00846456	I/II	Completed	GBM	20	DC vaccine targeting tumor stem cells + Radiotherapy + Temozolomide
NCT02313272	I	Completed	rGBM	32	Pembrolizumab + Hypofractionated stereotactic irradiation + Bevacizumab
NCT02617589	III	Completed	nGBM (Unmethylated MGMT)	560	Radiotherapy + Nivolumab VS Radiotherapy + Temozolomide
NCT02667587	III	Completed	nGBM (Methylated MGMT)	716	Radiotherapy + Temozolomide + Nivolumab VS Radiotherapy + Temozolomide + Placebo
NCT02829931	I	Completed	Recurrent high- grade gliomas	33	Nivolumab + Ipilimumab + Hypofractionated stereotactic irradiation + Bevacizumab
NCT02866747	I/II	Active	rGBM	108	Hypofractionated stereotactic radiotherapy + Durvalumab VS Hypofractionated stereotactic radiotherapy alone
NCT02968940	II	Completed	Transformed IDH Mutant GBM	6	Avelumab + Hypofractionated radiotherapy
NCT03047473	II	Completed	nGBM	30	Avelumab + Radiotherapy + Temozolomide
NCT03174197	I/II	Active	nGBM	80	Atezolizumab + Radiotherapy + Temozolomide
NCT03367715	II	Completed	nGBM (Unmethylated MGMT	10	Nivolumab + Ipilimumab + Radiotherapy
NCT03426891	I	Completed	nGBM	21	Vorinostat + Pembrolizumab + Radiotherapy + Temozolomide
NCT03576612	I	Active	nGBM	36	Gene mediated cytotoxic immunotherapy + Nivolumab + Radiotherapy + Temozolomide
NCT03661723	II	Completed	rGBM	60	Pembrolizumab + Radiotherapy
NCT03743662	II	Active	nGBM (Methylated MGMT)	39	Nivolumab + Radiotherapy + Bevacizumab
NCT04047706	I	Active	nGBM	18	Nivolumab + BMS-986205 (IDO1 inhibitor) + Radiotherapy + Temozolomide VS Nivolumab + BMS-986205 + Radiotherapy
NCT04396860	II/III	Active	nGBM (Unmethylated MGMT)	159	Ipilimumab + Nivolumab + Radiotherapy VS Radiotherapy + Temozolomide
NCT04729959	II	Recruiting	rGBM	53	Tocilizumab + Atezolizumab + Stereotactic radiotherapy
NCT04977375	I/II	Recruiting	rGBM	10	Pembrolizumab + Stereotactic radiotherapy
NCT05083754	I	Recruiting	nGBM	50	Carmustine wafer + Retifanlimab + Radiotherapy + Temozolomide VS Carmustine wafer + Retifanlimab + Radiotherapy

nGBM, new diagnosed glioblastoma; rGBM, recurrent glioblastoma; MGMT, O^6^-methylguanine (O^6^-MeG)-DNA methyltransferase.

The phase II trial (NCT02336165) assessed the effect of durvalumab in 5 GBM groups. The reported results were from group A, which evaluated durvalumab plus radiotherapy followed by durvalumab in nGBM with unmethylated MGMT promoter after undergoing the maximum safe surgical resection (86). The outcomes showed that combining durvalumab with radiotherapy was well tolerated and appeared to be effective for nGBM. Among 40 enrolled patients, 35% experienced grade 3 or higher TRAEs. Twenty-four patients (60%) were alive at 12 months, outperforming the historical benchmarks of 50%. The mOS was 15.1 months, outperforming the historical benchmarks of 12.7 months. Another phase I trial investigated the safety of combining durvalumab with hypofractionated stereotactic radiotherapy (HFSRT) in 6 rGBM patients (87). The outcomes showed that combining durvalumab with HFSRT in rGBM was well tolerated. Only 1 patient experienced grade 3 vestibular neuritis associated with durvalumab. The mOS and mPFS were 16.7 and 2.3 months, respectively. The relevant phase II trial is ongoing.

A phase I/II trial investigated the effect of atezolizumab combined with radiotherapy and temozolomide followed by adjuvant atezolizumab and temozolomide in nGBM (88). Sixty patients were enrolled. The primary endpoints were safety and OS, with secondary endpoints of ORR and PFS. Atezolizumab exhibited good tolerance and modest efficacy in combination with radiochemotherapy. Thirty-three patients experienced grade 3 or higher TRAEs. The mOS was 17.1 months, with a mPFS of 9.7 months.

The phase II trial (NCT03047473) investigated the effect of avelumab plus standard treatment of radiotherapy and temozolomide in nGBM (89). The results revealed that avelumab combined with standard treatment was safe. However, this combination treatment failed to improve the OS. Among 30 patients, 3 patients experienced emergent adverse events related to avelumab. And 8 patients experienced one or more iRAEs. The mOS was 15.3 months, with a mPFS of 9.7 months. The tolerability results in GBM population of this trial failed to exhibit novel safety signals in contrast to previous avelumab research. The ORR was superior compared to that observed with other immunotherapies for GBM. In addition to the different mechanisms of action of avelumab, it may also be attributed to the early administration of avelumab as an addition to standard treatment in the disease course.

The phase I trial (NCT02313272) evaluated the effect of pembrolizumab plus bevacizumab plus HFSRT for rGBM (90). A total of 32 patients were enrolled. The combination of pembrolizumab with bevacizumab and HFSRT triple therapy exhibited encouraging modest efficacy with well tolerance. No neurological adverse events were detected. Only one patient discontinued the treatment because of the AST elevation. The tumor response rates (complete response + partial response) in bevacizumab-naïve group (20 of 24 patients) and bevacizumab-resistant group (5 of 8 patients) were 83% and 62%, respectively. The mOS in bevacizumab-naïve group and bevacizumab-resistant group were 13.45 and 9.3 months, respectively. The mPFS were 7.92 and 6.54 months, respectively.

The recently completed phase I trial (NCT02311920) evaluated the effect of adding ipilimumab and nivolumab to radiochemotherapy for nGBM (91). The outcomes showed that ipilimumab plus nivolumab exhibited was well tolerated in combination with radiochemotherapy. Among 32 patients, 16% experienced grade 4 events. The mOS for patients treated with combined treatment was 20.7 months, with a mPFS of 16.1 months. Another phase I trial investigated ipilimumab and nivolumab in combined with HFSRT and bevacizumab for bevacizumab-naïve rGBM (92). The primary endpoint was tolerability of this combined treatment. The secondary endpoints were 6- and 9-months survival. This combined treatment was well tolerated. Among 26 patients, 12 experienced grade 3 or 4 TRAEs. The 6- and 9-months survival were 92% and 75%, respectively. The phase III trial (NCT02617589) compared nivolumab plus radiotherapy to temozolomide plus radiotherapy in nGBM (93). A total of 560 patients were evenly assigned into nivolumab plus radiotherapy group or radiotherapy plus temozolomide group. The outcomes showed that nivolumab plus radiotherapy failed to improve the OS compared to temozolomide plus radiotherapy, suggesting that nivolumab should not be considered a substitute for temozolomide in this patient population. The mOS of temozolomide group and nivolumab group were 14.9 and 13.4 months, respectively. The mPFS were 6.2 and 6.0 months, respectively. The response rates were 7.8% and 7.2%, respectively. The grade 3 or higher TRAE rates of nivolumab group and temozolomide group were 21.9% and 25.1%, respectively. Another phase III trial (NCT02667587) investigated the effect of Stupp regime plus nivolumab or placebo in nGBM (94). A total of 716 patients were evenly assigned into nivolumab + radiotherapy + temozolomide or placebo + radiotherapy + temozolomide. Unfortunately, nivolumab plus Stupp regime did not improve the survival. The mOS for nivolumab group and placebo group were 28.9 and 32.1 months, respectively. The mPFS were 10.6 and 10.3 months, respectively. For patients without baseline corticosteroids, the mOS of nivolumab group and placebo group were 31.3 months and 33.0 months. Moreover, nivolumab plus Stupp regime resulted in increased rates of TRAEs. The grade 3 or higher TRAE rates of nivolumab group and placebo group were 52.4% vs 33.6%, respectively. The neurological TRAEs were observed in 23.1% of nivolumab group and 16.7% of placebo group. The most TRAEs in both groups were headache (9.3%/5.9%) and dysgeusia (5.6%/4.2%). Lymphopenia rates were 10.7% and 8.5% in nivolumab group and placebo group, respectively.

In addition to ICIs, other immunotherapies combined with radiotherapy for GBM also has been investigated. A clinical trial investigated the effect of DC vaccine targeting tumor stem cells for GBM (95). The outcomes showed that DC vaccine against glioblastoma stem cells was well tolerated and improved the PFS. The mPFS for vaccinated patients was 2.9 times greater than control groups. Of the 7 patients treated with DC vaccine immunotherapy, all patients experienced immune response induced by vaccination without negative side effects.

## 6 Discussion

Immunotherapy combined with radiotherapy appears to be a promising therapeutic strategy, backed by a solid biological foundation. However, the complexities underlying their synergetic modes still require further understanding. There are still several current challenges that require to be addressed or better elucidated regarding the combination of immunotherapy and radiotherapy for GBM.

For example, radiotherapy boosts anti-tumor immune responses while also leading to immunosuppression. Severe lymphopenia is observed frequently in GBM patients after radiotherapy. A prospective study revealed that the decrease in CD4 lymphocyte counts was observed in nGBM patients received radiochemotherapy (96). And the decrease in CD4 counts was linked to tumor progression. A large irradiation area and excessive fractionation may impair the immune function (97). Therefore, decreasing dose to healthy tissues, reducing radiotherapy target volume and applying the hypofractionated radiotherapy regimen may limit this effect. In many tumors, Tregs have immunosuppressive effects or even facilitate the progression of disease. It has been demonstrated radiotherapy can increase significantly the number of Tregs by various mechanisms. For example, radiotherapy can facilitate the production, expansion, differentiation, and development of Tregs by overexpressing TGF-β. Tumor-associated neutrophils (TANs) are typically categorized into two types: N1 neutrophils, which are antitumorigenic, and N2 neutrophils, which are protumorigenic (98). Radiotherapy also prompts TANs to show pro-tumor properties by TGF-β (99). N2 neutrophils cause tumor growth and immunosuppression by suppressing multiple immune cells while increasing Tregs. Tumor-associated macrophages (TAMs) also are categorized into two types, M1 (anti-tumor phenotype) and M2 (pro-tumor phenotype) (100). The activated p50–p50 dimer and elevated ROS levels induced by radiotherapy can promote the shift to M2 macrophage, subsequently releasing IL-10 and TGF-β that suppress DCs (101). To utilize the synergistic effect between radiotherapy and immunotherapy more effectively, additional research is needed to investigate how to balance stimulating and suppressing effects caused by radiotherapy.

Another potential challenge in combining immunotherapy with radiotherapy for GBM is the potentiating treatment related effects, commonly known as pseudoprogression. It is defined as radiographic changes associated with tumor progression that are related to treatment response and are transient (102). Several studies have suggested that radiotherapy and temozolomide may result in pseudoprogression in GBM treatment. It also is reported in GBM patients who underwent immunotherapy (103). Therefore, combining immunotherapy with radiotherapy for GBM may result in stronger pseudoprogression. In addition to the diagnostic challenge, pseudoprogression also may bring treatment challenges, since medications commonly applied to manage symptoms related to pseudoprogression may affect the effectiveness of immunotherapy. While existing imaging technologies appear to exhibit partial capacity in distinguishing between pseudoprogression and tumor progression, there remains a considerable demand for improving diagnostic accuracy. Radiomics is a technique that utilizes data characterization algorithms to extract numerous features from medical images, serving as a promising method for distinguishing between different conditions. Kim et al. (104) created a radiomics model that utilizes multiparametric MRI and compared its diagnostic effectiveness for pseudoprogression with that for single parameter and single imaging radiomics models. The results showed that this model outperformed single radiomics models. Moreover, it exhibited better accuracy in external and internal validation than other single models. In another research, the accuracy, specificity, and sensitivity of 72.78%, 61.33%, and 78.36% of radiomics model in distinguishing pseudoprogression from tumor progression still outperformed the performance of three radiologists (105). These preliminary data demonstrated the potential of radiomics models in differentiating between pseudoprogression from tumor progression.

Moreover, ascertaining optimal schedules, such as immunotherapy types, radiation dose, fractionation regimen and treatment sequence when combining immunotherapy with radiotherapy, is necessary to exert maximally synergistic effect. Combining ICIs with radiotherapy for GBM has been investigated extensively. However, the study of other immunotherapies combined with radiotherapy for GBM is still limited. The efficacy of several vaccines and OVs for GBM has been demonstrated in clinical studies. Therapeutic vaccine combined with radiotherapy also has been demonstrated to improve the antitumor effect in experimental high-grade gliomas model. Given the current situation of combining ICIs with radiotherapy for GBM, exploring other immunotherapies combined with radiotherapy for GBM may be a feasible direction. Moreover, we need to consider the effect of radiotherapy dose and fractionation on immune response. Low dose radiotherapy (LDRT) has been demonstrated to mobilize efficiently immune system even when an immune suppression pathway is present (106). Conventional radiotherapy may induce immunosuppressive factors generation; however, LDRT could be a viable approach to overcome these challenges. LDRT also can promote T cells recruitment by increasing the expression of certain chemokines, subsequently enhancing the potential of abscopal effect. The immunomodulatory effects of LDRT have been demonstrated in preclinical and clinical studies. Therefore, we have reason to believe that LDRT may further improve the effect of the existing combined treatment of immunotherapy and radiotherapy. Further study is needed to evaluate comprehensively its potential in GBM. Hypofractionated radiotherapy, such as stereotactic body radiotherapy (SBRT), can administer higher dose in fewer sessions. Several studies have demonstrated that hypofractionated radiotherapy may be more effective in inducing immune responses (107, 108). A phase I trial evaluated the effect of hypofractionated radiotherapy combined with bevacizumab and pembrolizumab for rGBM (109). Among 32 patients, 53% experienced CR or PR. The 12-months survival rate was 64%. To achieve a better immunomodulatory effect, further randomized clinical trials are necessary to ascertain the ideal radiotherapy dose and fractionation.

Moreover, it has been widely recognized that the treatment sequence will affect significantly the therapeutic efficacy of combined treatment. However, the optimal sequence for immunotherapy combined with radiotherapy is currently unclear. A preclinical research reported that the group that administrated anti-PD-L1 during fractional radiotherapy resulted in a better survival outcome compared to the group that underwent sequential administration after finishing radiotherapy (110). Another preclinical research revealed that the group receiving anti-CTLA-4 prior to radiotherapy experienced a better tumor response compared to the group receiving anti-CTLA-4 after radiotherapy (111). Another retrospective review indicated that immunotherapy may be more effective when administrated simultaneously with or after radiotherapy (112). A clinical study compared the effect of neoadjuvant and concurrent immunotherapy to adjuvant immunotherapy in nGBM (113). Twenty-two patients underwent the vaccine before starting chemoradiation and continued during treatment (group 1). Twenty-three patients (group 2) underwent the vaccine after finishing chemoradiation. Patients in group 1 exhibited a stronger initial response before chemoradiation, but this response diminished during treatment. In contrast, patients in group 2 exhibited a weaker initial response, but this response was more durable. Based on these outcomes, the optimal treatment sequence may differ according to the immunotherapy used and tumor type. On the one hand, the treatment sequence of radiotherapy followed by immunotherapy enables immunotherapy to partially overcome the tumor tolerance to radiotherapy and improve the effectiveness of radiotherapy. On the other hand, the treatment sequence of immunotherapy followed by radiotherapy may help stimulate the immune microenvironment, such as normalizing the tumor vasculature, alleviating hypoxia in tumor, increasing the radiotherapy sensitivity of tumor. Further research is required to elucidate more accurately the effect of different sequences on therapeutic efficacy.

Combining immunotherapy with radiotherapy for GBM has performed well in preclinical studies, but its efficacy remains to be limited in clinical trials, which may be due to the following reasons. Firstly, the tumor growth environment and physiological characteristics of animal models are different from those of humans. It is difficult for animal models to fully simulate the complex heterogeneity of human GBM, resulting in a reduced effect of effective treatment in animal models in human. Secondly, certain inherent obstacles in GBM patients, such as intricate tumor microenvironment and tumor heterogeneity, may influence the effect of combination treatment. Glioblastoma cells are highly heterogeneous in different patients and even within the same tumor, which is difficult to fully cover in preclinical studies. In clinical practice, certain tumor cells might not respond well to combined treatment, which may affect overall therapeutic effect. Human GBM will create a complex immunosuppressive microenvironment, which can weaken the immune response activated by combination treatment. Although radiotherapy can break immune tolerance to a certain extent, it is difficult to completely change this immunosuppressive state. Moreover, the observational indicators and endpoints of preclinical studies are different from clinical studies. Preclinical studies pay more attention to short-term indicators such as tumor growth inhibition, while clinical studies pay more attention to long-term indicators such as OS, PFS, and quality of life. The transformation from short-term effect to long-term benefit is affected by various factors, resulting in the results of clinical studies being worse than expected.

Although combined treatment of immunotherapy and radiotherapy has demonstrated advantages in many tumors, only limited patients benefit from the combined treatment. Therefore, exploring accurate biomarkers to predict and evaluate therapeutic responses is necessary. Considering the rationale behind the combined treatment of immunotherapy and radiotherapy, the DAMPs may be potential biomarkers for predicting the effectiveness of combination treatment. Radiotherapy can result in ICD by increasing calreticulin. Furthermore, calreticulin induced by radiotherapy may be crucial in tumor cells uptake and enhancing immune cell activity (114). Therefore, the amount of calreticulin following radiotherapy may be a potential biomarker for combination treatment. The circulating lymphocyte population plays a crucial role in anti-tumor immune responses. There is evidence that higher lymphocyte counts are linked to increased response rate in patients receiving ICIs (115). Moreover, there is some evidence that radiosensitivity may predict the effect of combined treatment of immunotherapy and radiotherapy. A research evaluated the correlation between radiosensitivity and immune responses in various tumors, including GBM (116). The outcomes showed that radiosensitivity index-low tumors exhibited a higher proportion of activated NK cells and M1 macrophages compared to radiosensitivity index-high tumors.

Finally, there is evidence that alterations in gut microbiota and its metabolites may affect the development of various diseases, including GBM. Gut microbiota establishes connections between gut and central nervous system via two-way signals along gut–brain axis. Gut microbiota dysregulation can promote ROS pro advancement duction by downregulating GM-CSF signaling, subsequently enhances the suppressive effects of MDSCs (117). Furthermore, gut microbiota dysregulation may result in the growth and apoptosis inhibition of tumor cells by downregulating Foxp3 (118). Short-chain fatty acids (SCFAs) are the main metabolites of gut microbiota. They have been demonstrated to improve disease activity by increasing Tregs and reducing Th1 and Th17 cells (119). Overall, the immunosuppressive microenvironment of GBM closely related to gut microbiota. A deeper understanding of gut microbiota may provide new opportunities for GBM treatment.

## 7 Conclusion

Currently, the performance of immunotherapy in treating GBM is still not ideal due to the immunosuppressive microenvironment. Combining immunotherapy with radiotherapy has consistently been an active research area, and GBM is no exception. Although preclinical studies have demonstrated the efficacy of this combination treatment for GBM, relative clinical evidence is still limited. Combining immunotherapy with radiotherapy for GBM showed modest efficacy only in several clinical studies of limited size, there are no successful phase III trials all over the world to date. Further studies that include the tumor heterogeneity and microenvironment are required to expound the mechanisms of success or failure.
